# Burnout syndrome and resilience in medical students from a Brazilian public college in Salvador, Brazil

**DOI:** 10.47626/2237-6089-2020-0187

**Published:** 2021-06-01

**Authors:** Alan Roger Dias, Santiago Mozart Fernandes, Ivã Fialho-Silva, Thiago Cerqueira-Silva, Ângela Miranda-Scippa, Amanda Galvão-de Almeida

**Affiliations:** 1 Departamento de Neurociências e Saúde Mental Faculdade de Medicina de Bahia Universidade Federal da Bahia Salvador BA Brazil Departamento de Neurociências e Saúde Mental, Faculdade de Medicina de Bahia (FMB), Universidade Federal da Bahia (UFBA), Salvador, BA, Brazil.

**Keywords:** Burnout syndrome, medical students, resilience

## Abstract

**Introduction:**

Burnout syndrome is highly prevalent among medical students. Whereas burnout syndrome has been associated with negative outcomes, like suicidal ideation, protective factors are still unknown.

**Objective:**

To evaluate if there is an association between burnout syndrome and resilience in medical students, assessing covariates such as depressive symptoms, suicidal ideation, and religiosity.

**Method:**

This cross-sectional study was carried out with a sample of 209 students from a medical school in Brazil. Burnout syndrome was assessed using the Maslach Burnout Inventory – Student Survey. Potential protective factors and aggravators to burnout syndrome were investigated using appropriate scales.

**Results:**

Fifty-nine students (28.2%) presented burnout. Multivariate analysis showed that resilience was a protective factor (p < 0.001), along with being older, married or having better academic performance. Depressive symptoms were positively associated with burnout. Religiosity was not a protective factor and suicidal ideation was not associated with burnout when adjusted for depressive symptoms.

**Conclusion:**

Burnout is frequent among medical students, impacting mental health and academic performance. Resilience seems to be a protective factor, and the relationship between burnout and suicidal ideation is possibly mediated by depressive symptoms. Prospective studies are needed to further investigate the associations found in this study.

## Introduction

Burnout syndrome is a three-dimensional concept defined as a reaction to chronic emotional distress linked to work conditions.^1^ Among health care students, it is characterized by: 1) weariness in relation to academic activities; 2) disbelief and detachment from studies and patients; and 3) feeling of incompetence.^2^ Considering this definition, a systematic review with meta-analysis published in 2019 showed a 44.2% frequency of burnout in medical students around the world.^3^ This number has grown in the last decade and is notably increased in medical residency programs, reaching 60%, and suggesting that doctors possibly carry a load of burnout symptoms since graduation.^4^

A cross-sectional study showed that medical students, in general, have a higher frequency of burnout than students from other fields.^5^ Another study revealed strong associations between burnout and suicidal ideation (SI) in this population.^6^ However, it was shown that newly enrolled medical students have lower rates of burnout than students from the same period in other areas.^7^ This raises the hypothesis that, over the course of medical education, factors related to the academic context are added to individual predispositions, which together become responsible for the emergence of high levels of burnout throughout the medical program.^4^

From a different standpoint, support from family, friends, and colleagues, as well as high levels of religiosity/spirituality have been inversely related to the frequency of burnout in medical students.^8,9^ These findings are in line with what has already been proposed in the literature regarding the development of life-long resilience mechanisms, among which social support, including the relationship with family and community (e.g., active participation in a church, supportive friendships, marital relationship), is highly relevant among health protection factors.^10^

Recently, a systematic review has analyzed studies conducted in 43 countries and showed that at least one in nine medical students (11.2%) has SI and about 27.2% have depressive symptoms (DS),^11^ i.e., higher numbers than those described in the general population.^12^ In fact, many associations have been established with SI in medical students, and it is possible to synthesize them into two main groups: 1) factors related to the individual, such as heredity, personality characteristics, psychiatric morbidities,^13,14^ consumption of tobacco, alcohol, and other drugs^15^; and 2) factors related to the academic context, such as dissatisfaction with academic performance,^16^ perception of low performance, coming from other cities (being far from the family nucleus),^17^ and having thoughts of abandoning the medical program.^18^

Based on the above, we have hypothesized that: 1) the frequency of burnout is lower in medical students who have favorable mechanisms of resilience and greater religiosity; 2) the presence of burnout is associated with SI and DS.

## Methodology

### Study design and procedure

This was a cross-sectional study conducted with students from the Medical School of Bahia (Faculdade de Medicina de Bahia – Universidade Federal da Bahia [FMB-UFBA], Salvador, BA, Brazil). Data collection was carried out from September to October 2018. First, a pilot study (n = 25) was conducted using an online questionnaire to evaluate the respondent’s comprehension of the questions and to assess the time they spent completing them (approximately 19 minutes). The questionnaire was then redesigned after review of the items in which the participants had doubts. At the end of this stage, students were invited to answer an online questionnaire divided into 6 parts and were informed about the objectives of the research and the confidentiality of the data during the analysis.

This project was approved by the human research ethics committee of FMB-UFBA (project no. 2.764.716). In addition, based on Resolution 466/12, all participants were asked to read the informed consent form and, if they agreed, to sign and participate in the study.

### Sample

The sample consisted of FMB-UFBA medical students from all 12 semesters of the program. Inclusion criteria were: being 18 years old or older, being enrolled and regularly attending the FMB-UFBA. The exclusion criteria were incomplete responses or responses sent after the 1-month deadline informed on the platform.

### Instruments

#### Questionnaire on sociodemographic, academic, and psychophysiological data

This questionnaire covered the following topics: sex; age; self-declared ethnicity; marital status; religious affiliation; sexual orientation; birthplace; family income; if the respondent had work in addition to college; if they had children; cycle in the medical program (basic: 1st to 4th semester; clinical: 5th to 8th semester; internship: 9th to 12th semester); performance coefficient; participation in extracurricular activities; if they had ever stopped sleeping because of their studies; if they had already considered dropping out of the program; if they felt competitiveness; fear of failing; relationship with the professors; if death or poor prognosis of patients were a source of stress; if the program was a source of pleasure; if they were satisfied with the teaching strategy, with the skills acquired, and with their professional choice; and if they had already sought professional help for mental health complaints related to the program.

#### Maslach Burnout Inventory – Student Survey (MBI-SS)

This self-applicable questionnaire comprises 15 items with Likert-type answers ranging from 1 (never) to 6 (every day).^19^ It has been translated into and validated in Brazilian Portuguese.^2^ The MBI-SS has three dimensions: emotional exhaustion (items 1 to 5), cynicism (items 6 to 9), and professional efficacy (items 10 to 15). According to the sum of points in each dimension, burnout can be classified as low, moderate, or high. Low levels correspond to an emotional exhaustion total score of 0-9, cynicism 0-1, and professional efficacy > 27. Moderate levels correspond to emotional exhaustion 10-14, cynicism 2-6 and professional efficacy scores 23-27. High levels of burnout correspond to an emotional exhaustion score > 14, cynicism > 6, and professional efficacy < 23. We used the three-dimensional criteria (high score for emotional exhaustion and cynicism + low score for professional efficacy) to define the presence of burnout.

#### Beck Scale for Suicide Ideation (BSS)

The BSS is a 21-item self-administered questionnaire developed to assess the presence and severity of SI in the general population.^20^ It also evaluates the history of suicide attempts. Each item has three response options that vary on a scale from 0 to 2. No specific cut-off point has been defined in the literature to determine the absence or presence of SI; we considered that individuals scoring different than 0 had some degree of SI.

#### Patient Health Questionnaire 9 (PHQ-9)21

This questionnaire includes nine items based on criteria for major depression according to the fourth version of the Diagnostic and Statistical Manual of Mental Disorders (DSM-IV), with Likert responses ranging from 0 (never) to 3 (almost every day). The total score for PHQ-9 may range from 0 to 27; patients scoring ≥ 10 are considered to have DS. Regarding the severity of DS, the following classification is possible: absence (0-4); mild (5-9); moderate (10-14); moderate to severe (15-19); severe (> 20).

#### Wagnild and Young’s Resilience Scale (RS-25)

Validated for Brazilian Portuguese,^22^ the RS-25 has 25 items with Likert responses ranging from 1 (I totally disagree) to 7 (I totally agree), divided into five domains: serenity, perseverance, self-confidence, sense of life, and self-reliance. According to the sum of the scores of all items, the levels of resilience can be divided into high (score > 145), moderate (score between 125 and 145), or low (< 120).

#### Duke Religious Index (DUREL)

Developed by Koenig et al.^23^ and validated and translated into Brazilian Portuguese by Moreira-Almeida,^24^ DUREL measures the three dimensions of religious activity involved in health: organizational religious activity, which refers to the person’s attendance to religious meetings; non-organizational religious activity, which refers to the frequency of private religious activities (prayer, meditation, reading of religious texts, etc.); and intrinsic religiosity, which refers to the experience of religiosity as an individual’s goal. Higher scores on the DUREL scale indicate lower rates of religiosity in each of the three subdomains.

## Statistical analysis

The groups of students with and without burnout were compared in relation to the variables collected. The descriptive analysis of the continuous variables was performed using median and interquartile range (IQR) or mean and standard deviation (SD), as appropriate. Categorical variables were described as absolute and relative frequencies of the valid cases. The measure of association was expressed as prevalence ratio (PR) and 95% confidence interval (95%CI).

Multivariate analyses were conducted using a binary logistic regression model, after multiple imputation for all variables reaching p < 0.20 in the univariate analysis. Initially, a backward stepwise regression model was used including all pre-selected variables. Further, a hierarchical model was introduced including variables of clinical interest and those with p < 0.05 in the first model. For each variable included in the model, five individuals were needed in the lowest outcome group (burnout, n = 59), as proposed by Vittinghoff & McCulloch.^25^ The same methodology was applied to analyze the influence of DS and burnout in patients with SI. Data were analyzed with the Statistical Package for the Social Sciences (SPSS) version 21.0 for Windows.

## Results

The protocol was sent to 534 students via email, of which 100 responded completely; it was also shared via WhatsApp^®^ in class groups, which yielded another 109 complete responses (total of 209 students). Of the total sample, 59 students (28.2%) presented burnout. The mean age of the total sample was 23.8 (±4.0) years; 57.9% were women, 54.1% were brown skinned, 79.4% were heterosexual, and 53.6% declared to have some religion (Table 1).

**Table 1:** Sociodemographic, academic, and psychophysiological characteristics associated with burnout syndrome

Factor (n = 209)	Burnout		Overall sample	Prevalence ratio (95%CI)	p-value

	Yes (n = 59)	No (n = 150)			
Male	28 (47.5)	60 (40.0)	88 (42.1)	1.24 (0.81-1.91)	0.326
Age					
Mean (±SD)	23.2 (±3.7)	24.0 (±4.1)	23.8 (±4.0)	-	-
Median (IQR)	22 (21-25)	23 (21-26)	23 (21-26)	-	0.156
Self-declared ethnicity					
Brown skinned	36 (61.0)	77 (51.3)	113 (54.1)	1.41 (0.85-2.31)	
Black/indigene	6 (10.2)	15 (10.0)	21 (10.0)	1.26 (0.57-2.79)	0.390
White/yellow	17 (28.8)	58 (38.7)	75 (35.9)	Reference	
Single/other*	58 (98.3)	134 (89.3)	192 (91.9)	5.14 (0.76-34.8)	**0.045**
LGBT	13 (22.0)	30 (20.0)	43 (20.6)	1.09 (0.65-1.83)	0.734
Family income (US$)					
250-749	19 (32.2)	37 (24.7)	56 (26.8)	1.21 (0.71-2.06)	0.593
750-1,499	15 (25.4)	40 (26.7)	55 (26.3)	0.97 (0.54-1.74)	
1,500-2,499	7 (11.9)	27 (18.0)	34 (16.3)	0.73 (0.34-1.58)	
More than 2,500	18 (30.5)	46 (30.7)	64 (30.6)	Reference	
Working	9 (15.3)	20 (13.3)	29 (13.9)	1.17 (0.62-2.02)	0.718
Religious affiliation					
Catholic	14 (23.7)	41 (27.3)	55 (26.3)	0.80 (0.47-1.36)	0.756
Evangelical	8 (13.6)	19 (12.7)	27 (12.9)	0.93 (0.48-1.78)	
Spiritist/African origin religion/other	6 (10.2)	24 (16.0)	30 (14.3)	0.63 (0.29-1.35)	
No religion	31 (52.5)	66 (44.0)	97 (46.4)	Reference	
Medical program cycle					
Basic cycle	12 (20.3)	41 (27.3)	53 (25.4)	1.39 (0.62-3.10)	**0.020**
Clinical cycle	39 (66.1)	68 (45.3)	107 (51.2)	2.23 (1.13-4.1)	
Internship	8 (13.6)	41 (27.3)	49 (23.4)	Reference	
Performance coefficient (n = 179), %					
Mean (±SD)	7.7 (±1.0)	8.1 (±0.7)	8.0 (±0.8)	-	-
Median (IQR)	7.9 (7.5- 8.4)	8.1 (7.7-8.5)	8.0 (7.7-8.5)	-	**0.010**
Failing a course	17 (28.8)	21 (14.0)	38 (18.2)	1.82 (1.17-2.83)	**0.012**
Extracurricular activity	34 (57.6)	100 (66.7)	134 (64.1)	0.76 (0.49-1.17)	0.220
Sleep deprivation	45 (76.3)	106 (70.7)	151 (72.2)	1.23 (0.74 -2.07)	0.415
Thinking about giving up the program	43 (72.9)	62 (41.3)	105 (50.2)	2.66 (1.6-4.41)	**< 0.001**
Stressful relationship with teachers	50 (84.7)	114 (76.0)	164 (78.5)	1.52 (0.81-2.86)	0.166
Fear of failing academically	59 (100.0)	141 (94.0)	200 (95.7)	-	0.064
Stress due to poor patient prognosis	29 (49.2)	67 (44.7)	96 (45.9)	1.14 (0.74.-1.75)	0.558
Satisfaction with medical program	24 (40.7)	111 (74.0)	135 (64.6)	0.37 (0.24-0.58)	**< 0.001**
Satisfaction with professional choice	45 (78.9)	144 (97.3)	189 (92.2)	0.32 (0.22-0.46)	**< 0.001**
Satisfaction with teaching strategy	3 (5.1)	26 (17.4)	29 (13.9)	0.33 (0.11-0.99)	**0.020**
Satisfaction with the skills acquired	18 (30.5)	65 (43.9)	83 (56.1)	0.65 (0.41-1.06)	0.076

Data presented as n (%), unless otherwise specified.

95%CI = 95% confidence interval; IQR = interquartile range; LGBT = lesbians, gays, bisexual, transgender and transexual; SD = standard deviation.

* Compared to married or stable relationship.

### Relationship between burnout syndrome and resilience

Burnout was more frequent in students who showed low levels of resilience: 86.0 vs. 46.9%; PR = 4.65; p < 0.001 (Table 2).

**Table 2:** Relationship between burnout syndrome and suicidal ideation, resilience, depressive symptoms, and religiosity

Variable	Burnout		Overall sample	Prevalence ratio (95%CI)	p-value

	Yes (n = 59)	No (n = 150)			
Beck Scale for Suicide Ideation					
> 0	37 (62.7)	49 (32.7)	86 (41.2)	2.40 (1.53-3.77)	**< 0.001**
0	22 (37.3)	101 (67.3)	123 (58.8)	Reference	
Beck Scale for Suicide Ideation					
Mean (±SD)	6.52 (±8.43)	2.51 (±4.88)	3.64 (±6.34)	-	-
Median (IQR)	4 (0-10)	0 (0-2)	0 (0-6)	-	**< 0.001**
Suicide attempt (n = 205)					
Yes	6 (10.5)	9 (6.1)	15 (7.3)	1.49 (0.77-2.89)	0.368
No	51 (89.5)	139 (93.9)	190 (92.7)	Reference	
Resilience Scale (n = 200)					
High (> 145)	2 (3.5)	20 (14.4)	22 (11.0)	Reference	
Moderate (125-145)	6 (10.5)	56 (39.2)	62 (31.0)	1.06 (0.23-4.89)	**< 0.001**
Low (≤ 125)	49 (86.0)	67 (46.9)	116 (58.0)	4.65 (1.22-17.72)	
Resilience Scale (n = 200)					
Mean (±SD)	104.1 (±17.2)	125.0 (±18.6)	119.1 (±20.5)		-
Median (IQR)	126 (111-138)	104 (94-113)	118 (104-133)		**< 0.001**
Patient Health Questionnaire 9					
≥ 10	44 (74.6)	54 (36.2)	98 (47.1)	3.29 (1.96-5.53)	**< 0.001**
< 10	15 (25.4)	95 (63.8)	110 (52.8)	Reference	
Severity of depressive symptoms (n = 208)					
No MDD	7 (11.9)	45 (30.2)	52 (25.0)	Reference	**< 0.001**
Mild	8 (13.6)	50 (33.6)	58 (27.9)	1.03 (0.40-2.63)	
Moderate	16 (27.1)	25 (16.8)	41 (19.7)	2.90 (1.32-6.38)	
Moderate/severe	11 (18.6)	16 (10.7)	27 (13.0)	3.02 (1.33-6.91)	
Severe	17 (28.8)	13 (8.7)	30 (14.4)	4.20 (1.97-8.97)	
Organizational religious activity					
Mean (±SD)	4.88 (±1.48)	4.49 (±1.44)	4.60 (±1.46)	-	
Median (IQR)	5 (4-6)	5 (4-6)	5 (4-6)	-	**0.034**
Non-organizational religious activity					
Mean (±SD)	4.81 (±1.65)	4.28 (±1.73)	4.43 (±1.72)	-	-
Median (IQR)	6 (3-6)	5 (3-6)	5 (3-6)	-	**0.039**
Intrinsic religiosity					
Mean (±SD)	10.88 (±3.91)	9.41 (±3.98)	3.27 (±1.33)	-	-
Median (IQR)	11.5 (7.8-15.0)	10.0 (6.0-13.0)	10 (6.0-14.0)	-	**0.013**

Data presented as n (%), unless otherwise specified.

95%CI = 95% confidence interval; IQR = interquartile range; SD = standard deviation.

### Relationship between burnout syndrome and religiosity

No statistically significant differences were found between the groups regarding religious affiliation. However, in absolute numbers, a higher frequency of burnout was observed in students who declared not to have religion (52.5 vs. 44.0%). Students with burnout had higher scores in the three subdomains of DUREL, with the following mean results: organizational religious activity, 4.88 (±1.48) vs. 4.49 (±1.44), p = 0.034; non-organizational religious activity, 4.81 (±1.65) vs. 4.28 (±1.73), p = 0.039; and intrinsic religiosity, 10.88 (±3.91) vs. 9.41 (±3.98), p = 0.013 (Table 2).

### Relationship between burnout syndrome and suicidal ideation and depressive symptoms

Eighty-six students (41.1%) scored differently from 0 on BSS. Those with burnout had higher BSS scores when compared to those without the syndrome: 4 (0-10) vs. 0 (0-2), respectively (p = 0.001). Similarly, the frequency of SI was higher in those with burnout (62.7 vs. 32.7%; PR = 2.40; p < 0.001). There was a progressive increase in the prevalence of burnout as DS became more severe. Moderate to severe DS (PHQ-9 ≥ 10) were more frequent in participants with burnout (74.6 vs. 36.2%; PR = 4.29; p < 0.001) (Table 3).

**Table 3:** Binary logistic regression for burnout syndrome outcome

Variable	B	OR	95%CI for Exp(B)	p-value
Age	-0.165	0.848	0.799-0.899	**< 0.001**
Male	0.414	1.514	1.060-2.162	**0.023**
Being married	-1.828	0.161	0.059-0.439	**< 0.001**
Performance coefficient	-0.858	0.424	0.315-0.571	**< 0.001**
Thinking about dropping out of the program	0.812	2.253	1.582-3.207	**< 0.001**
No satisfaction with the program	1.359	3.892	2.732-5.544	**< 0.001**
Organizational religious activity	0.155	1.168	0.984-1.386	0.075
Non-organizational religious activity	-0.112	0.894	0.765-1.044	0.157
Intrinsic religiosity	0.003	1.003	0.927-1.086	0.940
Resilience	-0.058	0.944	0.934-0.954	**< 0.001**
Beck Scale for Suicide Ideation	0.001	1.001	0.974-1.028	0.948
Patient Health Questionnaire 9 > 10	0.710	2.034	1.416-2.922	**< 0.001**
Constant	14.586			

95%CI = 95% confidence interval; OR = odds ratio.

### Multivariate analysis

#### Factors associated with burnout syndrome

Table 4 shows the results of the binary logistic regression for SI, after adjustment for DS.

**Table 4:** Binary logistic regression for suicidal ideation outcome, adjusted for depressive symptoms (n = 207)

Variable	B	OR	95%CI for Exp(B)	p-value
Age	0.053	1.054	1.005-1.105	**0.031**
Male	-0.173	0.841	0.597-1.184	0.321
Family income (US$)				**< 0.001**
From 250 to 749	1.351	3.861	2.656-5.612	**< 0.001**
From 750 to 1,499	0.274	1.315	0.891-1.940	0.168
From 1,500 to 2,499	Reference	-	-	-
Resilience	-0.046	0.955	0.946-0.964	**< 0.001**
Burnout	-0.177	0.838	0.582-1.207	0.343
Semester	-0.142	0.868	0.817-0.922	**< 0.001**
Sexual orientation				**< 0.001**
Homosexual	0.175	1.191	0.709-2.002	0.509
Bisexual	1.735	5.668	3.426-9.375	**< 0.001**
Heterosexual	Reference	-	-	-
DUREL	0.004	1.005	0.862-1.170	0.954
Organizational religious activity				
Non-organizational religious activity	-0.008	0.992	0.866-1.137	0.913
Intrinsic religiosity	0.034	1.035	0.969-1.105	0.308
Sought professional help	1.108	3.028	2.155-4.254	**< 0.001**
Sleep deprivation	-0.388	0.679	0.476-0.967	0.032
PHQ-9 ≥ 10	1.260	3.526	2.540-4.896	**< 0.001**
Constant	4.766	117.485		**< 0.001**

95%CI = 95% confidence interval; DUREL = Duke Religious Index; OR = odds ratio; PHQ-9 = Patient Health Questionnaire 9.

The multivariate analysis conducted using binary logistic regression and having burnout as dependent variable showed that the variables male sex (odds ratio [OR] = 1.51; 95%CI 1.06-2.16), thinking about dropping out of the program (OR = 2.25; 95%CI 1.58-3.21), not having satisfaction with the program (OR = 3.89; 95%CI 2.73-5.54), and moderate to severe DS (OR = 2.03; 95%CI 1.41-2.92) were associated with higher chances of having burnout syndrome.

Other variables were associated with lower chances of having burnout, namely, being older (OR = 0.85; 95%CI 0.80-0.90), being married (OR = 0.16; 95%CI 0.06-0.44), having higher performance coefficient (OR = 0.42; 95%CI 0.32-0.57), and scoring higher in the RS-25 (OR = 0.94; 95%CI 0.93-0.95).

#### Relationship between burnout syndrome, suicidal ideation, and depressive symptoms

In a sensitivity analysis, we compared the occurrence of burnout as a predictor of SI in two different models: the first model without adjustment for DS (Table S1, available as online-only supplementary material), and the second model adjusted for DS (Table 4). In the first model, burnout behaved as an independent SI predictor (OR = 1.73; 95%CI 1.24-2.43; p < 0.001). However, after adjusting for DS, burnout lost its statistical significance (OR = 1.19; 95%CI 0.83-1.72; p < 0.343), whereas the occurrence of DS continued to behave as an independent predictor for SI (OR = 3.53; 95%CI 2.54-4.90; p < 0.001).

## Discussion

The present study found a prevalence of 28.2% of burnout in medical students at a federal university in the Northeast of Brazil, using the diagnostic criteria of MBI-SS. This result was lower than that found in a previously published meta-analysis (44.2%),^3^ which included 17,431 students from 24 studies conducted around the world. Conversely, in comparison with studies performed at universities located in other Brazilian states and using the same criteria, the frequency of burnout found in our sample was one of the highest reported: 10.3% was found in the state of Sergipe,^26^ 14.9% in Ceará,^27^ and 26.4% and 57.5% in two different studies conducted in São Paulo.^28,29^ These divergences may be due to different methodologies.

It is also worth mentioning that there is evidence that the frequency of burnout is higher in medical students than in students attending other undergraduate programs.^5^ Also, even though the problems reported by all university students are similar, their intensity, as experienced by medical students, seems to be higher.^30^ The relevance of this finding becomes more evident when taking into consideration the association between burnout and negative outcomes, such as less empathy, carelessness with the patient, medical errors and greater stigma in relation to mental health problems.^4^

Confirming our initial hypothesis, scoring higher on the RS-25 was associated with a lower chance of having burnout. In fact, we observed that students who had low or moderate levels of resilience had a prevalence of burnout about four times higher than those with high levels of resilience, which demonstrates the protective factor of this variable in this population. In this sense, it is reasonable to conclude that students who have a greater ability to adapt to negative situations also tend to cope better with stress. However, in comparison with the general population, the literature points out that medical students have lower levels of resilience,^31^ which would make them more vulnerable, possibly due to a lack of psychological resources to confront or limit the effect of stressors.^32^

Thus, it becomes important to provide this population with opportunities to increase their levels resilience, so as to improve their quality of life, medical education, and, ultimately, patient care.^33^ According to the model that explains the dynamics of resilience among medical students, proposed by Dunn et al., even though resilience is an individual characteristic, it can be encouraged by the educational institution in several ways, e.g., by developing individual plans to improve academic performance with study strategies, offering empathic support for struggling students, and ensuring that those at risk receive appropriate professional support.^32^

In relation to religiosity, although the students without burnout presented higher levels of religiosity, the potential protective effect of this variable was not maintained in the multivariate analysis. In our sample, such effect was more moderate when compared to the findings of another study, in which a different scale was used to measure religiosity; that study reported that religiosity was a protective factor against burnout in this population.^34^

We also found that older students presented lower chances of having burnout. We hypothesized that older individuals would be better prepared to face exposure to adverse events over time and would therefore be more resilient and present less burnout. This may have contributed to a natural selection scenario, in which only those who were able to adapt remained in the program. Unfortunately, we did not investigate dropout rates, and neither did we perform any follow-up of the participants. It is also important to mention that, in our sample, higher levels of resilience were not associated with increased age.

Better academic performance measured by the performance coefficient remained as a protective factor against the development of burnout. However, due to the cross-sectional nature of our study, we cannot state whether students with this syndrome had lower performance also because of the condition, or whether the lower academic performance would predispose students to higher levels of burnout. The same applies to the program being a source of satisfaction vs. having thoughts of dropping out of the program – both associated with burnout.

Being male was associated with a higher chance of developing burnout, which is in accordance with the literature.^35^ Being single was associated about five times higher chances of having burnout when compared with being married. Moreover, being married was an independent predictor of protection. It is possible that factors related to relationship stability, such as emotional support of the partner, are related to lower levels of burnout.

Students with lower income (from US$ 250 to 749) presented a higher prevalence of burnout, which might be explained by the stress resulting from the impossibility of helping in the family livelihood, since only a minority of the students could coordinate work and studies.

Students in the clinical cycle (5th to 8th semester) presented a higher frequency of burnout when compared to internship and basic cycle students. We raised three hypotheses to explain this finding. The first hypothesis concerns the opposition between the students’ expectations and the reality regarding the initial contact with patients and the public health system. This moment can be frustrating when the limitations of medical treatment are perceived in the face of chronic, incurable, or often lethal conditions.^36^ The second hypothesis is that, in the basic cycle, satisfaction with the students’ recent approval in the college admission exam would still predominate, whereas the internship period would be characterized by excitement with the proximity of graduation. Conversely, the clinical cycle would be an intermediate stage, in which the anxiety so far accumulated is further combined with concerns about the future.^37^ Third, contact with the patient in a context of inexperience, which is characteristic of the clinical cycle, could predispose students to greater anxiety and a greater risk of developing burnout.^37^

Similarly, having failed a discipline, fear of failing in the academic context, dissatisfaction with professional choice and with teaching strategies were more frequent in students with burnout. The association of burnout with all these variables had already been demonstrated in the literature.^26,27^ Even with the impossibility of establishing causal relationships, our results strongly suggest an association between this syndrome and negative aspects of both the academic and personal life of the student.

Observable factors in the daily life of a medical student, such as the constant tension generated by excessive demands for better results, either by the individual himself or by society,^36^ abusive power relations by some teachers,^37^ outdated evaluation systems with low levels of evidence regarding effectiveness,^4^ and a routine that involves sacrifice of leisure,^28^ demonstrate plausibility of the results found. However, the possibility that personality traits with more anankastic characteristics and higher levels of neuroticism may negatively interfere with the outcome of burnout^38^ should not be discarded. In fact, almost half (45%) of the students reported having already sought professional assistance for some psychological demand related to the medical program.

As for the relationship between burnout and SI, in our sample, having some degree, although minimum, of SI in the last week was associated with a 2.4 times higher frequency of burnout. However, in the multivariate analysis, burnout did not remain associated with SI when adjusted for DS and other variables (Table 4). This confirms our initial hypothesis, namely, that the higher prevalence of burnout among students with SI may be due to the presence of DS.

In this sense, a multicenter American study involving 4,287 medical students showed that burnout could be an independent predictor for SI,^6^ this being the worst outcome that burnout could have. However, there is published evidence of the association of SI with other variables, such as personality characteristics,^13^ consumption of tobacco, alcohol and other drugs,^15^ and psychiatric morbidities,^14^ which can influence the association found in our study.

In our sample, the frequency of moderate to severe DS was 47.1%, much higher than the numbers found in a previous meta-analysis involving medical students (27.2%),^12^ and was an independent predictor of burnout (OR = 2.03; 95%CI 1.42-2.92; p < 0.001). This result is ratified in the literature, which also shows that burnout is responsible for the maintenance of DS throughout the medical degree program.^39^

Knowing that DS is one of the main predictor variables of SI among university students,^40^ we researched whether this could be a confounding factor in the burnout × SI relationship. In fact, our findings suggest that much of the SI found among students with burnout is a result of the presence of DS, since this condition behaved as an independent predictor of SI (OR = 3.53; 95%CI 2.54-4.90; p < 0.001) (Table 4).

### Limitations

Our main limitations are: the cross-sectional study design, which does not allow to assess causality; the use of self-applicable scales, naturally with limited precision; having considered only the finished questionnaires and not the entire classes, generating possible selection bias, which may overestimate the frequencies found; and finally, the reduced size sample (n = 209) if compared to other Brazilian studies on burnout.^27-29^ Moreover, because of differences in curriculum content and teaching methodology across the several medical schools available in the world, caution is needed when trying to extrapolate our results.

### Strengths

To the authors’ knowledge, this is the first study to evaluate the relationship between SI and burnout among medical students in Brazil. It is also one of the first to evaluate variables such as religiosity and resilience through scales, in the context of burnout.

## Final considerations

Burnout is a frequent problem that has important consequences in terms of mental health and academic performance in the population assessed. Having greater resilience levels proved to be a protective factor against burnout. Burnout did not prove to be an independent predictor of SI when adjusted for DS in the multivariate analysis. Likewise, religiosity did not prove to be a protective factor. Therefore, it is suggested that interventions with the potential to increase resilience be prioritized by medical college institutions to reduce burnout among their students, in addition to providing reception services for students with DS.

## Supplementary material


