# Supplementary material for: Burnout syndrome and resilience in medical students from a Brazilian public college in Salvador, Brazil
Source: Trends Psychiatry Psychother. 2021 Jun 1;44:e20200187. doi: 10.47626/2237-6089-2020-0187 (PMC9907392; doi:10.47626/2237-6089-2020-0187)
Supplement: Supplementary file 1 [file 2238-0019-trends-44-e20200187-suppl01.pdf]

http://dx.doi.org/10.47626/2237-6089-2020-0187

Table S1 - Binary logistic regression for suicidal ideation outcome, not adjusted for depressive symptoms (n = 207)

**Variable**	**B**	**OR**	**95%CI for Exp(B)**	**p-value**
Age	0.058	0.943	0.902-0.986	**0.010**
Male gender	-0.148	1.160	0.832-1.617	0.382
Family income (U$)				**< 0.001**
250-749	1.342	0.261	0.182-0.375	**< 0.001**
750-1,499	0.127	0.881	0.610-1.273	0.500
1,500-2,499	Reference	-	-	-
Resilience	-0.049	1.051	1.041-1.060	**< 0.001**
Burnout	0.550	1.733	1.237-2.430	**< 0.001**
Semester	0.164	1.178	1.112-1.248	**< 0.001**
Sexual orientation				
Heterosexual	1.389	4.010	2.494-6.445	**< 0.001**
Homosexual	1.339	3.815	2.007-7.253	**< 0.001**
Bisexual	Reference	-	-	-
DUREL				
Organizational religious activity	0.016	1.017	0.876-1.180	0.829
Non-organizational religious activity	0.025	1.025	0.899-1.168	0.713
Intrinsic religiosity	-0.041	0.960	0.901-1.023	0.210
Sought professional help	1.316	0.268	0.194-0.372	**< 0.001**
Sleep deprivation	-0.157	1.169	0.830-1.648	**< 0.001**
Constant	-5.653	0.004		**< 0.001**

95%CI = 95% confidence interval; OR = odds ratio.
